# LncRNA XIST promotes neovascularization in diabetic retinopathy by regulating miR-101-3p/VEGFA

**DOI:** 10.20945/2359-4292-2023-0097

**Published:** 2024-05-10

**Authors:** Weina Fu, Yunyan Ye, Feng Hu

**Affiliations:** 1 Ningbo University Ningbo Medical Center Lihuili Hospital Department of Ophthalmology Ningbo P.R. China Department of Ophthalmology, Ningbo Medical Center Lihuili Hospital, Ningbo University, Ningbo, P.R. China

**Keywords:** Diabetes, retinopathy, lncRNA XIST, miR-101-3p, VEGFA, angiogenesis

## Abstract

**Objective:**

This study sought to investigate the regulation of long noncoding RNA (lncRNA) XIST on the microRNA (miR)-101-3p/vascular endothelial growth factor A (VEGFA) axis in neovascularization in diabetic retinopathy (DR).

**Materials and methods:**

Serum of patients with DR was extracted for the analysis of XIST, miR-101-3p, and VEGFA expression levels. High glucose (HG)-insulted HRMECs and DR model rats were treated with lentiviral vectors. MTT, transwell, and tube formation assays were performed to evaluate cell viability, migration, and angiogenesis, and ELISA was conducted to detect the levels of inflammatory cytokines. Dual-luciferase reporter, RIP, and RNA pull-down experiments were used to validate the relationships among XIST, miR-101-3p, and VEGFA.

**Results:**

XIST and VEGFA were upregulated and miR-101-3p was downregulated in serum from patients with DR. XIST knockdown inhibited proliferation, migration, vessel tube formation, and inflammatory response in HG-treated HRMECs, whereas the above effects were nullified by miR-101-3p inhibition or VEGFA overexpression. miR-101-3p could bind to XIST and VEGFA. XIST promoted DR development in rats by regulating the miR-101-3p/VEGFA axis.

**Conclusions:**

LncRNA XIST promotes VEGFA expression by downregulating miR-101-3p, thereby stimulating angiogenesis and inflammatory response in DR.

## INTRODUCTION

Diabetic retinopathy (DR) is a microvascular complication of diabetes, leading to specific changes in the fundus of the eye (1). The global prevalence of DR is reported to be 22.27% among patients with diabetes in 2019 (2). When DR progresses at the proliferative (advanced) stage, the retina develops new blood vessels, resulting in vitreous/pre-retinal hemorrhage and tractional retinal detachment (3). With regard to the pathology, hyperglycemia-induced metabolic abnormalities stimulate oxidative stress, which causes inflammation and regulates neovascularization in the retina (4). Vascular endothelial growth factor (VEGF) is known as an established molecule in the pathogenesis of DR and intravitreal administration of agents antagonizing VEGF shows favorable outcomes in the management of DR (5). VEGF exerts a vital role in mediating the activities of several kinases and ultimately drives cell proliferation, migration, and vascular permeability during vascularization (6). A comprehensive and profound understanding of the complicated mechanisms underlying the regulation of VEGF may provide a basis for optimizing the therapeutic effect of anti-VEGF therapy on DR.

The human genome expresses a mass of long noncoding RNAs (lncRNAs) that confer diverse roles in cell biology and human disease at epigenetic, transcriptional, and translational levels (7). Recent investigation has come to light indicating the role of several lncRNAs in regulating endothelial function in DR, such as MEG3, VEAL2, and MALAT1 (8-10). LncRNA X inactive-specific transcript (XIST), which is transcribed from the *XIST* gene responsible for X chromosome inactivation, is shown to regulate retinal glial and epithelial cells under hyperglycemia (11,12). Moreover, lncRNA XIST maintains VEGF signaling in human brain microvascular endothelial cells and is required for hypoxia-induced angiogenesis (13). However, the role of XIST in human retinal microvascular endothelial cells (HRMECs) in DR remains unclear. It is well-acknowledged that lncRNAs can interact with microRNAs (miRNAs/miRs) to regulate downstream mRNA and gene expression (14). XIST targets miR-101-3p and inhibits its expression in silicosis, cancers, and bronchopulmonary dysplasia (15-18). Interestingly, miR-101-3p can repress vascular endothelial growth factor A (VEGFA) expression by targeting VEGFA mRNA, thus reducing cell migration and invasion (19).

In light of the above evidence, we hypothesized that XIST might maintain VEGFA expression by targeting miR-101-3p, thereby driving retinal angiogenesis in DR. This study was conducted to dissect the function of the XIST/miR-101-3p/VEGFA axis in DR for the purpose of uncovering new molecular mechanisms underlying the pathogenesis of DR, hoping to provide a reliable theoretical basis for exploring potential targets for the treatment of DR.

## MATERIALS AND METHODS

### Clinical samples

This study enrolled 20 patients with DR (13 males and 7 females, aged 59.21 ± 9.57 years) who visited the Department of Ophthalmology in Ningbo Medical Center Lihuili Hospital from August 2021 to January 2022. Additionally, another 20 patients with diabetes but without retinopathy (11 males and 9 females, aged 56.11 ± 9.79 years) were included. The basic characteristics of all patients are listed in Table 1. The patients with DR were included according to the WHO diagnostic criteria for diabetes (20) and diagnostic criteria for DR (21). None of the participants had cardiovascular diseases, peripheral vascular diseases, liver or kidney dysfunction, or malignancy. Venous blood samples were collected after 12-h fasting. This study was approved by the Ethics Committee of Ningbo Medical Center Lihuili Hospital and totally complied with the Declaration of Helsinki. All patients had signed the written informed consent.

**Table 1:** Basic characteristics of patients

	Non-DR (n = 20)	DR (n = 20)
Age (years)	56.11 ± 9.79	59.21 ± 9.57
Male/female	11/9	13/7
Disease course (years)	8.05 ± 2.35	8.29 ± 2.67
BMI (kg/m2)	22.8 ± 1.75	23.32 ± 1.15
FBG (mmol/L)	6.81 ± 1.62	7.49 ± 1.76*
HbA1c (%)	7.65 ± 2.04	12.61 ± 1.35*

Abbreviations: DR, diabetic retinopathy; BMI, body mass index; FBG, fasting blood glucose; HbA1c, hemoglobin A1c.

* *p* < 0.05, *vs*. non-DR.

### Cell culture and treatment

Primary HRMECs (MIC-iCell-m009; iCell Bioscience, Shanghai, China) were cultured at 37 °C with 5% CO_2_ in human endothelial cell medium (ScienCell, Carlsbad, CA, USA) comprising 10% FBS (Thermo Fisher Scientific, Wilmington, DE, USA), 100 U/mL penicillin (Gibco, Grand Island, NY, USA), 100 μg/mL streptomycin (Gibco), and 1% endothelial cell growth supplement (Lonza, Walkersville, MD, USA). The cellular models of DR were established as previously described (22). The third-generation HRMECs were randomized to the control and high glucose (HG) groups once attaining approximately 80% confluence. The control group was cultured with 5.5 mM glucose (Sigma-Aldrich, St. Louis, MO, USA), while the HG group was treated with 25 mM glucose for 48 h.

### Cell transfection and grouping

XIST knockdown short hairpin RNA (shRNA) vector (sh-XIST), miR-101-3p inhibitor (in-miR-101-3p), XIST overexpression vector (oe-XIST), miR-101-3p mimic (mi-miR-101-3p), VEGFA overexpression vector (oe-VEGFA), and corresponding negative controls (NCs) were procured from Hanbio (Shanghai, China). Following 48-h co-transfection of HEK293 cells with lentiviral vectors, lentiviral particles in the cell supernatant were separated by centrifugation at 500 × *g* for 10 min and filtration. HRMECs were infected with the lentiviruses for subsequent experiments.

### qRT-PCR

The isolation of total RNA was conducted with TRIzol and the concentration was subsequently determined using a spectrometer (DU-640; Beckman, San Jose, CA, USA). The RT kits (RR047A; Takara, Japan) and miRNA first strand cDNA synthesis (tailing reaction) kits (B532451-0020; Sangon, Shanghai, China) were used to synthesize cDNA for detection of mRNA and miRNA, respectively. Gene expression was examined using SYBR Green Mix (HY-K0501A; MedChemExpress, Monmouth Junction, NJ, USA) and LightCycler 480, with a reaction volume of 20 μL and the following parameters: 95 °C (10 s); 30 cycles of 95 °C (5 s), 60 °C (10 s), and 72 °C (10 s); and finally 72 °C (5 min). Data analysis was performed with the 2^−ΔΔCt^ method (each sample was tested in triplicate). PCR primers are shown in Table 2.

**Table 2:** PCR primers and their sequences

Primers	Sequences
LncRNA XIST-F-homo	CTTGGATGGGTTGCCAGCTA
LncRNA XIST-R-homo	TCATGCCCCATCTCCACCTA
LncRNA XIST-F-rattus	CAGCCTCGGTCTCTCGAATC
LncRNA XIST-R-rattus	CTTGGTGGCCAGGATGGAAT
miR-101-3p-F-homo	CATCGCACGTACAGTACTGTGATA
miR-101-3p-R-homo	CTCTGTCTCTCGTCTTGTTGGTAT
miR-101-3p-F-rattus	TCCGAAAGTCAATAGTGTC
miR-101-3p-R-rattus	GTGCAGGGTCCGAGGT
U6-F-homo	CGCTTCGGCAGCACATATAC
U6-R-homo	TTCACGAATTTGCGTGTCATC
U6-F-rattus	CTCGCTTCGGCAGCACA
U6-R-rattus	AACGCTTCACGAATTTGCGT
VEGFA-F-homo	TCACCAAGGCCAGCACATAG
VEGFA-R-homo	GAGGCTCCAGGGCATTAGAC
VEGFA-F-rattus	CGACAGAAGGGGAGCAGAAA
VEGFA-R-rattus	GCTGGCTTTGGTGAGGTTTG
GAPDH-F-homo	AATGGGCAGCCGTTAGGAAA
GAPDH-R-homo	GCGCCCAATACGACCAAATC
GAPDH-F-rattus	ACCACAGTCCATGCCATCAC
GAPDH-R-rattus	TCCACCACCCTGTTGCTGTA

Abbreviations: F, forward primer; R, reverse primer; homo, human gene; rattus, rat gene.

### Western blotting

Protein samples were mixed with the loading buffer (Beyotime, Shanghai, China) and heated for 3 min. After electrophoresis (0.5 h at 80 V and 1-2 h at 120 V), the proteins were moved to the membranes (300 mA, 60 min). After rinsing, the membranes were soaked overnight in blocking buffer, followed by 1-h treatment with primary antibodies including anti-VEGFA (ab1316), anti-occludin (ab216327), anti-GAPDH (ab8245; as the reference) (all 1:1000; Abcam, Cambridge, MA, USA), anti-ZO-1 (sc-33725, 1:500; Santa Cruz, CA, USA), and anti-claudin-5 (ABT45, 1:1000; Sigma-Aldrich) and then 1-h re-probing with horseradish peroxidase (HRP)-tagged anti-IgG (A0208, 1:1000; Beyotime). A chromogenic reagent was dripped onto the membranes, and the protein expression was detected and analyzed with a chemiluminescence imaging system (Bio-Rad, Hercules, CA, USA) and Quantity One software 4.6.2.

### MTT detection of cell viability

Cells in 96-well plates (3 wells/sample) were treated with MTT solution (10 μL/well, M6494; Thermo Fisher Scientific) for 120 min. The reaction was terminated by adding dimethyl sulfoxide (Sigma-Aldrich). The absorbance at 450 nm was measured with the help of a Bio-Rad microplate reader (model 680).

### Transwell detection of cell migration

Cells in the exponential growth stage were trypsinized and then diluted to a concentration of 1 × 10^5^ cells/mL with the 1% FBS-complete medium. Cell suspension (200 μL) was transferred to each upper chamber of a 24-well plate equipped with transwell inserts, and 10% FBS-medium (500 μL) was added to the lower chamber. After 48-h culture, non-invasive cells were removed, and the insert membrane was washed twice with PBS. Invasive cells were treated with 5% paraformaldehyde and 0.5% crystal violet for 15 min each, and the number of cells passing across the membrane was counted under an Olympus microscope (CKX53).

### Tube formation assay

The assay was carried out as previously described (23). Matrigel (Corning, Tewksbury, MA, USA) was pre-dissolved at 4°C, and 96-well plates (Millipore, Billerica, MA, USA) and pipette tips were pre-chilled. Each well was plated with 100 μL of Matrigel, and the plate was then placed in an incubator for 30 min to solidify the Matrigel. HRMECs were seeded in the 96-well plates pre-coated with Matrigel (2 × 10^4^ cells/well, 3 replicate wells/sample). The cells were routinely cultured for 18 h and photographed under a CKX53 microscope. A tube was considered a tubular structure that extended from one branching point to another or to a loose end. Capillaries in 3 visual fields/group were counted using Image-Pro Plus 6.0 software.

### ELISA

The levels of TNF-α, IL-6, and IL-1β were detected in conformity with the provided manuals of ELISA kits (R&D Systems, UK). Samples were pre-incubated in an ELISA plate (96 wells) overnight at an ambient temperature. Thereafter, 100 μL of 5% BSA was added to each well to block nonspecific binding for 60 min. The samples were incubated with the primary antibody (diluted in 5% BSA-PBS, 100 μL/well) for 3 h and next with the HRP-labeled secondary antibody (diluted in 5% BSA-PBS, 100 μL/well) for 60 min. After washing with PBS, the plate was incubated with 10 μL of substrate for 10-15 min, followed by measurement of absorbance_450 nm_.

### Dual-luciferase reporter assay

The binding sites between miR-101-3p with lncRNA XIST and VEGFA were predicted by starBase (http://starbase.sysu.edu.cn/). A wild-type sequence (wt-XIST or wt-VEGFA) or mutant-type sequence (mut-XIST or mut-VEGFA) of the predicted binding sites was cloned to the pGL3-Basic and co-delivered with 30 nM miR-101-3p or NC mimic into the 293T cells. Following 48-h transfection, luciferase activity was detected using a detection kit (Promega, Madison, WI, USA) and analyzed using a Promega system.

### RNA pull-down

The Pierce^TM^ magnetic RNA-protein pull-down kit (Millipore) was utilized in our assay. Biotinylated NC or miR-101-3p probes (Geneseed, Guangzhou, China) were incubated with cell lysates for 120 min at 25 °C. Immune complexes were pulled down by streptavidin-tagged immunomagnetic beads and treated in proteinase K buffer for 60 min at 25 °C respectively, followed by qRT-PCR analysis of the eluted RNA.

### RNA immunoprecipitation (RIP)

Magnetic beads were suspended in the RIP wash buffer (100 μL) and kept with anti-Ago2 (ab186733) or anti-IgG (ab172730) antibody (both 1:100 and 5 μg; Abcam) at an ambient temperature for 0.5 h. The bead tube was placed on a magnetic base, and then the supernatant was removed. The complexes were washed twice with RIP wash buffer (500 μL). The prepared bead tube was placed on the magnetic base, the supernatant was removed, and RIP immunoprecipitation buffer (900 μL) was added. The complexes were kept with cell lysate 100 (μL) overnight. After a brief spin, magnetic precipitation, and six washes, the complexes were incubated with proteinase K buffer 150 (μL) for 0.5 h at 55 °C. The tube was then put on the magnetic base, and the supernatant was extracted for qRT-PCR RNA detection.

### Establishment of a DR rat model

Thirty male Sprague-Dawley (SD) rats (200 ± 20 g; Beijing Vital River, China) were kept in the specific-pathogen-free animal room (21-25 °C, 50%-65% relative humidity, and 12 h/12 h light-dark cycles), with free access to water and food. Animal experiments were performed after the animals adapted to the environment for 7 days, with permission by the ethics committee of Ningbo Medical Center Lihuili Hospital. Streptozotocin (STZ; S0130, 65 mg/kg; Sigma-Aldrich) in citrate buffer (0.01 M, pH 4.5) was intraperitoneally injected twice (on days 1 and 4, respectively) into rats on an empty stomach (12-h fasting before each injection) to cause diabetes. Control rats were injected twice with an equal amount of citrate buffer. Blood glucose levels were detected every 2 days after STZ injection. Rats were considered diabetic when their blood glucose levels exceeded 16.7 mmol/L.

### Animal grouping and treatment

The 30 SD rats were grouped as control, DR, sh-XIST+oe-NC, sh-XIST+oe-VEGFA, and sh-NC+oe-NC. In the latter three groups, 1 μL of lentiviruses (10° PFU/mL (24,25)) as indicated in their group names were injected into the vitreous cavity of rats on the 7th day after successful induction of diabetes. Subsequent experiments were performed 4 weeks after lentiviral injection.

### Hematoxylin-eosin (H&E) staining of retinal tissue

Rats were anesthetized by IP injection of 1% pentobarbital sodium (40 mg/kg). The eyeballs were collected with ophthalmic scissors, the anterior segment of the left eye was excised, and retinal tissue was isolated and fixed with 4% paraformaldehyde (4 °C, overnight). The retina and sclera were dehydrated with graded ethanol, paraffin-embedded, and sliced (5 μm in thickness). After deparaffinization and rehydration, the sections were sequentially treated with hematoxylin (3-5 min), 1% hydrochloric acid alcohol (20 s), 1% ammonia water (30 s), and eosin (5 min). After dehydration (75%, 90%, 95%, and 100% ethanol) and clearing (xylene), the samples were mounted and observed under an Olympus microscope.

### Statistical analyses

Statistics were analyzed with GraphPad Prism 8, and all data were depicted as mean ± SD. All cell experiments were conducted in triplicate. Two groups were compared using t-test, and one-way analysis of variance was implemented for comparisons among three or more groups, followed by Tukey's multiple comparisons test. *P* < 0.05 represented a statistically significant difference.

## RESULTS

### Low XIST expression inhibits angiogenesis and inflammatory response in HG-treated HRMECs

**Figure 1:**
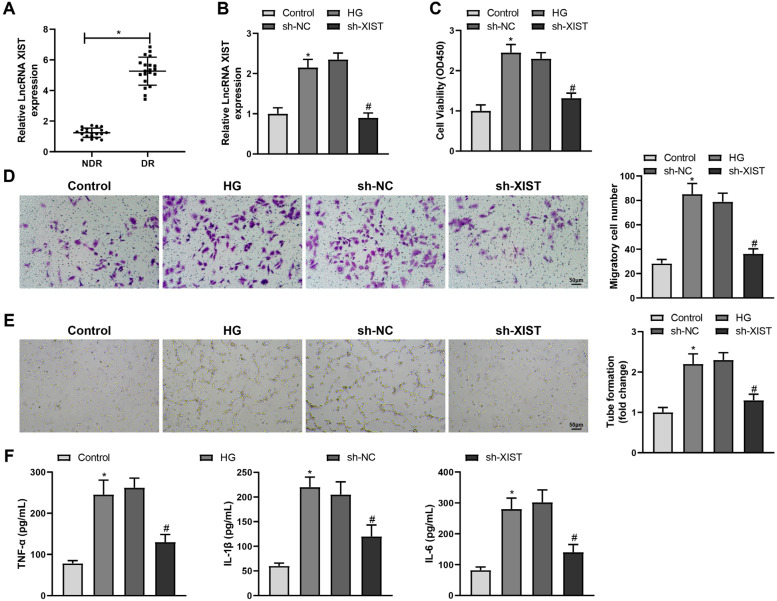
Low expression of XIST inhibits angiogenesis and inflammatory response in HG-treated HRMECs. (**A**) qRT-PCR was used to detect XIST expression in patients with diabetes or DR (n = 20). HRMECs were treated with HG and transfected with sh-NC or sh-XIST. Next, (**B**) qRT-PCR was performed to detect XIST expression, (**C**) MTT assay to detect cell proliferation, (**D**) transwell assay to detect cell migration, (**E**) tube formation assay to detect angiogenesis, and (**F**) ELISA to detect levels of inflammatory factors in culture supernatant. The data were expressed as mean ± standard deviation. Each cell experiment was repeated thrice. ^*^P < 0.05, compared with the NDR or control group; ^#^*P* < 0.05, compared with the sh-NC group.

First, qRT-PCR was carried out to detect XIST expression in patients with diabetes or DR, which revealed remarkable upregulation of XIST expression in patients with DR (Figure 1A, ^*^P < 0.05). Consistently, XIST expression was increased in HG-treated HRMECs, the cellular models of DR (Figure 1B, ^*^P < 0.05). Next, to examine the function of XIST in DR, the silencing vector sh-XIST was utilized to induce low expression of XIST in HG-treated HRMECs (Figure 1B, ^#^*P* < 0.05). MTT and transwell assays were conducted to assess cell viability and migration, respectively, which showed that HG treatment stimulated the proliferation and migration of HRMECs (Figure 1C, D, ^*^P < 0.05). Tube formation assay revealed that HG treatment increased the angiogenic ability of HRMECs (Figure 1E, ^*^P < 0.05). However, the proliferative, migratory, and angiogenic abilities of HG-treated HRMECs were weakened by XIST downregulation (Figure 1C-E, ^#^*P* < 0.05). Moreover, ELISA detection showed that HG treatment elevated the levels of TNF-α, IL-1β, and IL-6 in HRMEC culture supernatant (Figure 1F, ^*^P < 0.05). The levels of these cytokines declined upon sh-XIST interference (Figure 1F, ^#^*P* < 0.05). Consequently, downregulation of XIST inhibits angiogenesis and inflammatory response in HG-treated HRMECs.

### XIST targets miR-101-3p in DR

**Figure 2:**
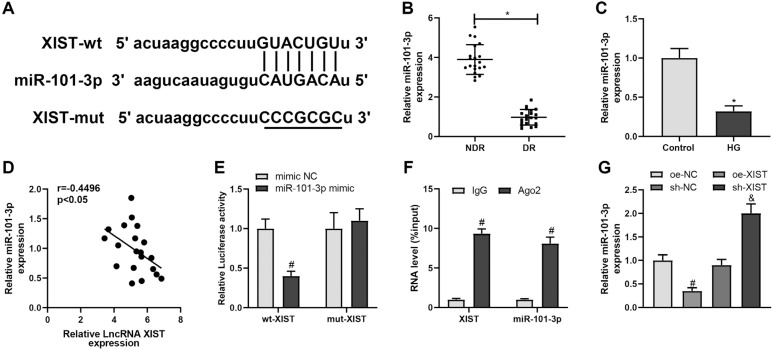
XIST targets miR-101-3p in DR. (**A**) The binding sites and corresponding mutations of miR-101-3p and XIST. qRT-PCR was used to detect miR-101-3p expression in patients with DR (**B**) and HG-treated HRMECs (**C**). (**D**) Pearson's correlation analysis of the expression of XIST and miR-101-3p in patients with DR. Dual-luciferase reporter (**E**) and RIP (**F**) assays were performed to verify the binding of miR-101-3p and XIST. (**G**) qRT-PCR was used to detect the expression of miR-101-3p in HRMECs transfected with oe-NC, oe-XIST, sh-NC, or sh-XIST. The data were expressed as mean ± standard deviation. The clinical sample size was 20. Each cell experiment was repeated thrice. ^*^P < 0.05, compared with the NDR or control group; ^#^*P* < 0.05, compared with the mi-NC, oe-NC, or IgG group; ^&^*P* < 0.05, compared with the sh-NC group.

The starBase database predicted the binding sites between XIST and miR-101-3p (Figure 2A). Therefore, we hypothesized that XIST may regulate angiogenesis and inflammatory response in HRMECs via miR-101-3p. qRT-PCR detection showed that miR-101-3p expression was reduced in both patients with DR and HG-treated HRMECs (Figure 2B, C, ^*^P < 0.05). In addition, the Pearson's correlation analysis indicated a significant negative correlation between XIST and miR-101-3p expression in patients with DR (Figure 2D). Next, dual-luciferase reporter and RIP assays were conducted to validate the interaction between XIST and miR-101-3p. Specifically, mi-miR-101-3p significantly diminished the luciferase activity of the reporter vector containing wt-XIST (Figure 2E, ^#^*P* < 0.05). The anti-Ago2 antibody captured a significant amount of XIST as well as miR-101-3p (Figure 2F, ^#^*P* < 0.05). Further, the results unveiled that miR-101-3p was notably downregulated in HRMECs upon oe-XIST transfection and upregulated after XIST silencing (Figure 2G, both *P* < 0.05). Overall, these data indicate that XIST targets and negatively regulates miR-101-3p in DR.

### miR-101-3p downregulation in HG-treated HRMECs reverses anti-angiogenic and anti-inflammatory effects of XIST knockdown

**Figure 3:**
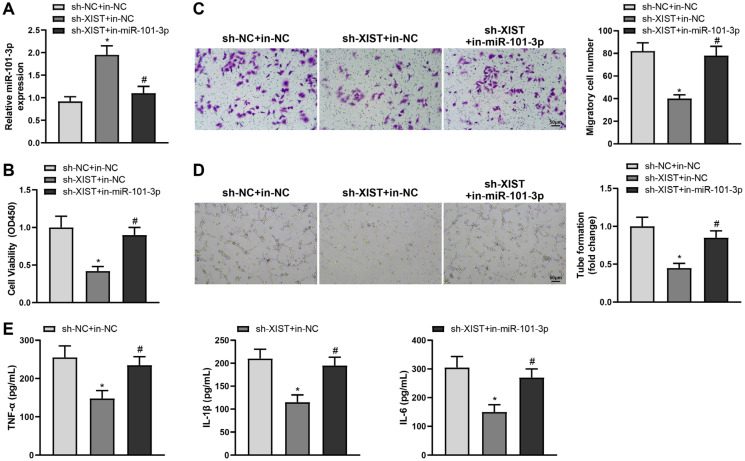
Downregulation of miR-101-3p reverses anti-angiogenic and anti-inflammatory effects of XIST knockdown on HG-treated HRMECs. HRMECs were treated with HG and transfected with sh-NC + in-NC, sh-XIST + in-NC, or sh-XIST + in-miR-101-3p. Next, (**A**) qRT-PCR was performed to detect miR-101-3p expression, (**B**) MTT assay to detect cell proliferation, (**C**) transwell assay to detect cell migration, (**D**) tube formation assay to detect angiogenesis, and (**E**) ELISA to detect levels of inflammatory factors in culture supernatant. The data were expressed as mean ± standard deviation. Each cell experiment was repeated thrice. ^*^P < 0.05, compared with the sh-NC+in-NC group; ^#^*P* < 0.05, compared with the sh-XIST+in-NC group.

To clarify the involvement of the XIST/miR-101-3p axis in HRMEC angiogenesis and inflammatory response, sh-XIST and in-miR-101-3p were delivered into HG-treated HRMECs and subsequently qRT-PCR validated the transfection effectiveness of in-miR-101-3p (Figure 3A, ^#^*P* < 0.05). The viability, migration, and tube forming activities of HG-insulted HRMECs were evidently weakened in the sh-XIST + in-NC group relative to that in the sh-NC + in-NC group (^*^P < 0.05) but potentiated in the sh-XIST + in-miR-101-3p group compared to that in the sh-XIST + in-NC group (Figure 3B-D, ^#^*P* < 0.05). ELISA revealed that TNF-α, IL-1β, and IL-6 levels were diminished in the sh-XIST + in-NC group (^*^P < 0.05) but increased in the sh-XIST + in-miR-101-3p group (Figure 3E, ^#^*P* < 0.05). In summary, XIST promotes angiogenesis and inflammatory response in HG-treated HRMECs by downregulating miR-101-3p.

### VEGFA is targeted by miR-101-3p in DR

**Figure 4:**
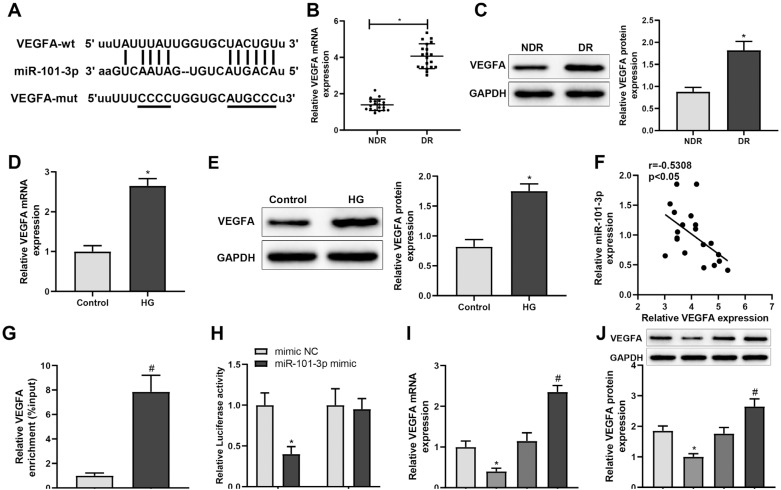
miR-101-3p targets VEGFA in DR. (**A**) The miR-101-3p-binding site in VEGFA and corresponding mutations. qRT-PCR and western blotting were used to detect the mRNA expression and protein level of VEGFA in patients with DR (**B, C**) and HG-treated HRMECs (**D, E**). (**F**) Pearson's correlation analysis of the expression of VEGFA and miR-101-3p in patients with DR. RNA pull-down (**G**) and dual-luciferase reporter (**H**) assays were performed to verify the binding of miR-101-3p and VEGFA. qRT-PCR (**I**) and western blotting (**J**) were used to detect the mRNA expression and protein level of VEGFA in HRMECs transfected with mi-NC, mi-miR-101-3p, in-NC, or in-miR-101-3p. The data were expressed as mean ± standard deviation. The clinical sample size was 20. Each cell experiment was repeated thrice. ^*^P < 0.05, compared with the NDR, control, or mi-NC group; ^#^*P* < 0.05, compared with the NC probe or in-NC group.

The TargetScan database predicted the binding sites in VFGFA for miR-101-3p (Figure 4A). Therefore, we hypothesized that XIST may regulate VFGFA via miR-101-3p to affect angiogenesis and inflammatory response in HRMECs. We found that the mRNA and protein levels of VFGFA were elevated in patients with DR as well as HG-treated HRMECs (Figure 4B-E, ^*^P < 0.05). Moreover, miR-101-3p and VFGFA expression levels were inversely correlated in patients with DR (Figure 4F). Next, RNA pull-down and dual-luciferase reporter assays were performed to validate the relationship between miR-101-3p and VFGFA. Specifically, biotinylated miR-101-3p probes captured VFGFA (Figure 4G, ^#^*P* < 0.05). mi-miR-101-3p significantly lowered the luciferase activity of the reporter vector containing wt-VFGFA (Figure 4H, ^*^P < 0.05). To further verify the relationship between miR-101-3p and VFGFA in HRMECs, VFGFA expression in HRMECs transfected with mi-NC, mi-miR-101-3p, in-NC, or in-miR-101-3p was determined, respectively. VFGFA expression was reduced upon mi-miR-101-3p transfection but elevated by in-miR-101-3p transfection, respectively (Figure 4I, J, both *P* < 0.05). Taken together, the data indicate that miR-101-3p targets and negatively regulates VFGFA in DR.

### VFGFA overexpression reverses anti-angiogenic and anti-inflammatory effects of XIST knockdown on HG-treated HRMECs

**Figure 5:**
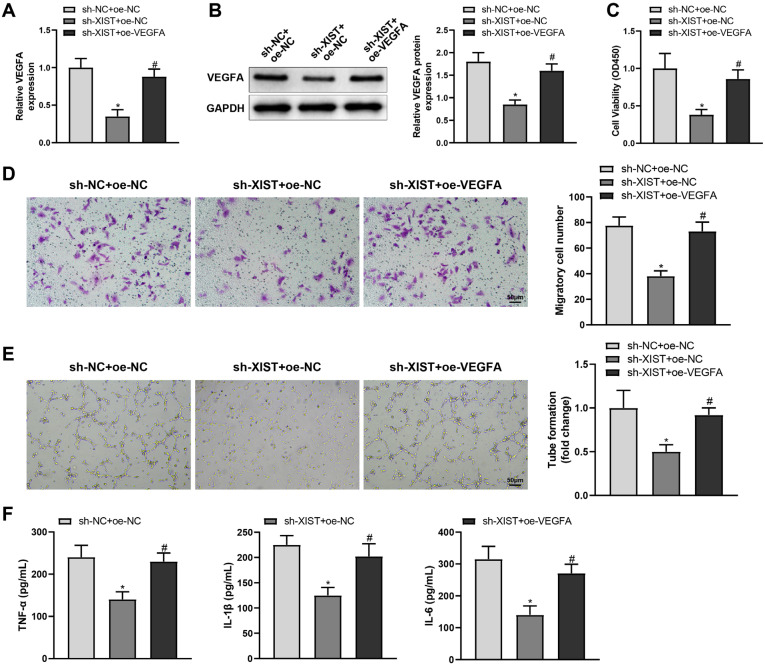
VFGFA overexpression reverses the anti-angiogenic and anti-inflammatory effects of XIST knockdown on HG-treated HRMECs. HRMECs were treated with HG and transfected with sh-NC + oe-NC, sh-XIST + oe-NC, or sh-XIST + oe-VFGFA. Next, (**A**) qRT-PCR was performed to detect VFGFA mRNA expression, (**B**) western blotting to detect VFGFA protein expression, (**C**) MTT assay to detect cell proliferation, (**D**) transwell assay to detect cell migration, (**E**) tube formation assay to detect angiogenesis, and (**F**) ELISA to detect levels of inflammatory factors in culture supernatant. The data were expressed as mean ± standard deviation. Each cell experiment was repeated thrice. ^*^P < 0.05, compared with the sh-NC+oe-NC group; ^#^*P* < 0.05, compared with the sh-XIST+oe-NC group.

To confirm whether XIST regulates the miR-101-3p/VFGFA axis to affect HRMEC angiogenesis and inflammatory response, sh-XIST and oe-VEGFA were delivered into HG-treated HRMECs. sh-XIST transfection decreased the expression of VEGFA (^*^P < 0.05). Under sh-XIST transfection, the expression of VEGFA was partially restored by oe-VEGFA transfection (Figure 5A, B, ^#^*P* < 0.05). Moreover, VFGFA overexpression reversed the anti-angiogenic and anti-inflammatory effects of XIST knockdown on HG-treated HRMECs (Figure 5C-F, ^#^*P* < 0.05). Therefore, XIST stimulates angiogenesis and inflammatory response in HG-treated HRMECs by increasing VFGFA expression.

### XIST promotes retinal angiogenesis and inflammatory response in rats with DR through the miR-101-3p/VEGFA axis

**Figure 6:**
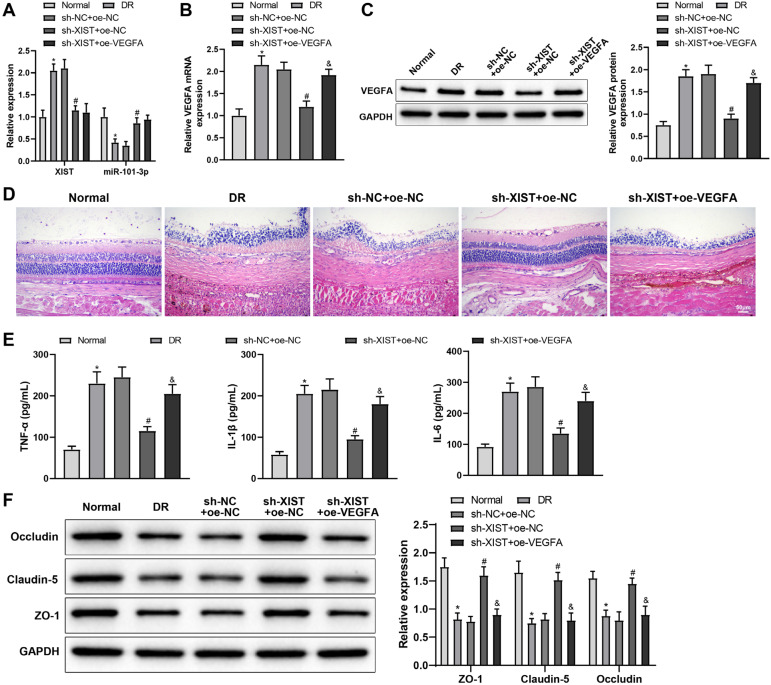
XIST promotes retinal angiogenesis and inflammatory response in rats with DR through the miR-101-3p/VEGFA axis. DR model rats were injected with sh-NC + oe-NC, sh-XIST + oe-NC, or sh-XIST + oe-VFGFA. (**A, B**) qRT-PCR was used to detect the expression levels of XIST, miR-101-3p, and VEGFA mRNA in retinal tissue. (**C**) Western blotting was used to detect the expression of VEGFA protein in retinal tissue. (**D**) H&E staining was used to detect pathological changes in the retina. (**E**) ELISA was performed to detect the levels of TNF-α, IL-1β, and IL-6 in retinal tissue. (**F**) Western blotting was used to detect the expression levels of ZO-1, claudin-5, and occluding in retinal tissue. The data were represented by mean ± standard deviation. Each group had 6 rats. ^*^P < 0.05, compared with the normal group; ^#^*P* < 0.05, compared with the sh-NC+oe-NC group; ^&^*P* < 0.05, compared with the sh-XIST+oe-NC group.

Finally, to clarify the regulation of the XIST/miR-101-3p/VEGFA axis in DR *in vivo*, DR model rats were injected with sh-XIST + oe-NC, sh-XIST + oe-VFGFA, or sh-NC + oe-NC. The expression levels of XIST and VEGFA were increased and miR-101-3p was downregulated in the retinal tissue of DR model rats (Figure 6A-C, ^*^P < 0.05). H&E staining and ELISA were used to detect pathological changes and pro-inflammatory factor levels in retinal tissue. The inner limiting membrane of the retina of control rats was smooth, without structural abnormalities, and the retinal layers were clear in structure and normal in thickness. The outer layer of the retina of DR model rats exhibited distorted morphology and reduced thickness (Figure 6D). DR model rats also showed higher levels of IL-6, TNF-α, and IL-1β than control rats (Figure 6E, ^*^P < 0.05). sh-XIST injection repressed XIST and VEGFA expression, promoted miR-101-3p expression, and reduced the pathological changes and pro-inflammatory factor levels in the retinal tissue of DR model rats (Figure 6A-E, ^#^*P* < 0.05). sh-XIST-induced inhibition of VEGFA expression, alleviation of histopathological changes, and reduction of retinal inflammation were counteracted by oe-VEGFA (Figure 6A-E, ^&^*P* < 0.05). In addition, the expression levels of permeability-related proteins ZO-1, claudin-5, and occludin in retinal tissue were measured utilizing western blotting, which were decreased in DR model rats (^*^P < 0.05) but increased after sh-XIST treatment (^#^*P* < 0.05). The upregulation of these proteins caused by XIST knockdown was nullified by VEGFA overexpression (Figure 6F, ^&^*P* < 0.05). Altogether, XIST promotes retinal angiogenesis and inflammatory response in rats with DR by modulating miR-101-3p/VEGFA.

## DISCUSSION

The number of people with DR keeps rising worldwide with the increasing prevalence of diabetes and prolonged lifetime of patients with diabetes (26). VEGFA is regarded as a predominant pathogenic factor in regulating neovascularization in DR, and intravitreal administration of anti-VEGF molecules has radically improved the management of DR (27). Increasing the duration of anti-VEGF therapy and developing more optimized molecules are hot issues in this field. This study elucidated upregulated XIST and VEGFA and downregulated miR-101-3p in patients with DR. XIST indirectly upregulated VEGFA by targeting miR-101-3p, thereby stimulating angiogenesis in HG-treated HRMECs.

Many studies have been conducted to address the mechanisms underlying the regulation of VEGF in DR. For example, berberine, an isoquinoline alkaloid, is known to inactivate the Akt/mTOR signaling in retinal endothelial cells to inhibit insulin-induced activity of hypoxia-inducible factor-1α and VEGF (28). miR-21 activates the phosphatidylinositiol 3-kinase/Akt/VEGF signaling pathway by targeting phosphatase and tensin homolog, thereby stimulating vascular endothelial cell viability and angiogenesis in the retina of rats with diabetes (29). Circular RNA COL1A2 contributes to HG-induced proliferation, migration, and vascular permeability in HRMECs and retinal angiogenesis in mice with diabetes by competing with VEGF mRNA for binding to miR-29b (30). There are many other miRNAs, such as miR-15b, miR-23a, miR-205-5p, and miR-150-5p, which directly interact with VEGF in retinal endothelial cells and are involved in angiogenesis in DR (31-34). This study identified miR-101-3p as a direct regulator of VEGFA in HRMECs.

As previously reported, miR-101-3p suppresses the release of VEGFA from cancer-associated fibroblasts by targeting VEGFA mRNA and subsequently reduces migration and invasion of non-small cell lung cancer cells (19). Although the function of miR-101-3p in HRMECs has not been reported to date, there are several studies indicating the implications of miR-101-3p for vascular endothelial function. For example, compelling evidence suggests that miR-101-3p overexpression promotes ROS production and induces cytoskeletal destruction in human umbilical vein endothelial cells by targeting TET2 (35). Zika virus could upregulate hsa-miR-101-3p in human brain microvascular endothelial cells to suppress VE-cadherin and claudin-5, two endothelial barrier integrity-responsible factors (36). Moreover, miR-101-3p expression is downregulated to promote endothelial-mesenchymal transition and renal fibrosis in mice with diabetes (37), suggesting the involvement of miR-101-3p in the regulation of endothelial function in diabetes. Many lncRNA-miRNA interplays are involved in the development of DR (38). Consistent with previous findings about the interaction of lncRNA XIST with miR-101-3p (16,17,39), this study established the XIST/miR-101-3p axis in HRMECs.

Current knowledge further indicates that HG-induced downregulation of XIST increases apoptosis and reduces migration in human retinal pigment epithelial cells by upregulating hsa-miR-21-5p expression (11). Moreover, XIST is downregulated to facilitate HG-induced activation of retinal Müller cells and production of pro-inflammatory cytokines (12). However, evidence favoring the function of XIST in HRMECs in DR is sparse. Wang and cols. have found that XIST silencing impedes angiogenesis and exacerbates cerebral vascular injury in ischemic stroke by regulating proangiogenic integrin α5 and anti-inflammatory Krüppel-like transcription factor 4 via miR-92a (40). In addition, XIST upregulation protects brain microvascular endothelial cells from pyroptosis following ischemic injury (41). In this study, low XIST expression was uncovered to repress proliferation, migration, angiogenesis, and secretion of pro-inflammatory cytokines in HG-treated HRMECs.

Accumulating evidence has suggested that VEGFA can be regulated by the lncRNA/miRNA network in DR. For instance, linc00174 could deteriorate diabetic retinal microangiopathy by modulating the miR-150-5p/VEGFA pathway (33). Malat1 knockdown suppresses the release of VEGFA by targeting miR-205-5p to curb HRMEC growth and tube formation under HG conditions (32). Mechanistically, TUG1 acts as a competing endogenous RNA for miR-145 to promote VEGFA expression in HRMECs, and repression of miR-145 annuls the beneficial roles of TUG1 silencing in HG-stimulated HRMECs (42). However, there is no report regarding the action of the XIST/miR-101-3p/VEGFA axis in HRMECs. Therefore, we then conducted a series of experiments with anticipation to fill this knowledge gap. Similar to the previously reported lncRNA/miRNA/VEGFA axis, our results elucidated that downregulation of miR-101-3p or overexpression of VEGFA neutralized the anti-angiogenic and anti-inflammatory effects of XIST knockdown on HG-treated HRMECs. An existing research has pointed out the constant high abundance of miR-101 in developing rat retinas (43), suggesting the essential role of miR-101 in the development of retinas. Intrinsically, the upregulation of VEGFA is shown to disrupt the retinal barrier, contributing to chronic damage to the neurovascular structure of the retina, ultimately causing vision loss (44). VEGFA overexpression confers pro-migratory, pro-proliferative, and pro-angiogenic actions in HG-treated HRMECs (45,46).

Subsequently, animal experiments were performed for *in vivo* verification of this mechanism. As well-known, disruption of the blood-retinal barrier (BRB) is the pathophysiological basis for increased vascular permeability and macular edema in DR and is an important cause of vision loss in patients with DR (47). BRB integrity is determined by junctional complexes composed of tight junctions and adherent junctions, and cell-to-cell connection is composed of tight junctions, adherent junctions, and desmosomes, among which tight junctions are responsible for the barrier function between cells (48). The tight junctions are the basis of the BRB and are essential for maintaining the structural and functional integrity of the BRB as well as the stability of the intraretinal environment. The BRB tight junctions are mainly composed of transmembrane proteins, cytoplasmic adhesion proteins, and cytoskeletal proteins. The transmembrane proteins are mainly composed of claudins, occludins, and ZO-1 (48). Therefore, the expression levels of permeability-related proteins (ZO-1, claudin-5, and occludin) can be determined to evaluate retinal lesions. Consistent with the findings obtained from cell experiments, downregulation of XIST reduced the histopathological changes and levels of TNF-α, IL-1β, IL-6, and meanwhile upregulated ZO-1, claudin-5, and occludin levels in the retina of rats with DR, but the above effects were counteracted by VEGFA overexpression. Collectively, the aforementioned findings and evidence underscore that XIST drives angiogenesis and inflammatory response in DR by upregulating VEGFA expression via miR-101-3p.

This study is the first to clarify the function of XIST and miR-101-3p in HRMECs in DR. The findings that VEGFA is regulated by the XIST/miR-101-3p axis provide a valuable reference for the treatment of DR and a novel direction for further investigations. Our research reveals that XIST facilitates VEGFA expression by downregulating miR-101-3p, thus stimulating angiogenesis and inflammatory responses in DR, indicating the potential roles of XIST and miR-101-3p as promising targets of anti-VEGFA therapy for DR. However, one limitation of this work is the relatively small sample size of included patients. Additionally, exploring the mediation of other XIST-regulated miRNAs in VEGFA is worthwhile. The downstream pathways of VEGFA in DR also deserve further investigations to comprehensively understand the mechanisms underlying anti-VEGFA therapy. Moreover, further research of preclinical aspect is warranted to prompt the clinical use of anti-VEGFA therapy in the management and treatment of DR.
